# Marijuana-Induced Acute Hepatitis: A Case Report

**DOI:** 10.7759/cureus.30273

**Published:** 2022-10-13

**Authors:** Hazem Abosheaishaa, Mahmoud Nassar, Muhammad Haseeb ul Rasool, Karim Makhoul, Mohammed Abdelwahed

**Affiliations:** 1 Internal Medicine, Icahn School of Medicine at Mount Sinai/NYC Health+Hospitals/Queens, New York City, USA; 2 Internal Medicine/Gastroenterology, Cairo University, Giza, EGY; 3 Medicine, Icahn School of Medicine at Mount Sinai/NYC Health+Hospitals/Queens, New York City, USA; 4 Pathology, Donald and Barbara Zucker School of Medicine at Hofstra/Northwell, Hempstead, USA

**Keywords:** acute fulminant hepatitis, drug induced hepatitis, drugs, marijuana, hepatitis

## Abstract

Marijuana is among the most widely used recreational drugs in the United States. The most common side effects of marijuana include mood changes, impaired memory, impaired body movements, and hallucination. Chronic use of marijuana is associated with transaminitis and hepatomegaly. Reported cases of acute hepatitis secondary to heavy marijuana smoking are very rare.

## Introduction

Hepatitis is characterized by liver inflammation and is caused by infectious pathogens, medications, autoimmune diseases, environmental toxins, alcohol, or illicit drugs [1]. In studies of chronic marijuana users, 76.9% had transaminitis, 57.7% had hepatomegaly, 73.1% had splenomegaly, and 46.2% had hepatosplenomegaly [1]. Alanine aminotransferase levels increased in healthy people who received therapeutic doses of cannabidiol after 3.5 weeks [2]. Here, we present a case with acute hepatitis subsequent to marijuana use.

## Case presentation

A 34-year-old female African American with a history of polysubstance abuse, including the use of alcohol and marijuana, presented to the emergency department with persistent nausea and vomiting. The patient was an active smoker, unemployed, and undomiciled. The patient reported multiple episodes of non-bloody, non-bilious vomiting over the past two days without identifying a precipitating factor. She was unable to quantify the amount of vomiting. She tried drinking water and soda, but it always came back up. The patient reported feeling hot, chills, intermittent shortness of breath, generalized weakness, and right hypochondrial discomfort with back pain for one day. She stated that she last consumed alcohol three days ago. The patient denied any use of IV drugs or recent travel but admitted to smoking cigarettes and marijuana. When asked about the usual amount that she used to smoke, she responded "whenever I get some, I smoke all of them"; she stated that she normally smoked more than 10 marijuana joints before feeling weak. Her previous surgical history includes the repair of an inguinal hernia on the right side. There is no history of drug allergies. There are no home medications she takes. Previously, she was hospitalized for anemia caused by the uterine fibroid, requiring blood transfusions. There is no history of liver disease in the patient's family. The review of other systems was unremarkable.

The patient's vital signs indicated that she had a blood pressure of 146/94 mmHg, a heart rate of 78 beats per minute, a temperature of 98.5 F, and a saturation of 98%. Physical examination revealed dry mucous membranes and mild tenderness of the liver (palpated 2 cm below the costal margin). A basic blood test at the emergency department revealed significant elevations in liver function tests (LFTs) as in Table 1. The urine was positive for tetrahydrocannabinol (THC), and the alcohol level was negative. 

**Table 1:** Laboratory results ALP: alkaline phosphatase; ALT: alanine transaminase; AST: aspartate transaminase; INR: international normalized ratio; Hb: haemoglobin; HCT: hemocrit; PLT: platelet count; Na: sodium; K: potassium; BUN: blood urea nitrogen; PCO2: partial pressure of carbon dioxide; HCO3: bicarbonate; CK: creatine kinase

Laboratory test	On presentation	Day 1	Day 2	Day 3	Reference range
Albumin	5.1	4.4	3.7	4.1	3.5-5.5 g/dl
Bilirubin	0.6	0.4	0.2	<0.2	0.0-0.3 mg/dl
ALP	411	363	262	209	35-104 U/L
ALT	411	319	189	101	0-33 U/L
AST	2636	1787	726	168	5-35 U/L
INR	1.1	1.1	11		1-1.1
Hb	9.3				12-14.5 g/dl
HCT:	32.9				37.0 - 47.0 %
WBCs	6.86				4-10.5 x10^3^/mcl
PLT	199				150-450 x10^3^/mcl
Na	133				136-145 mmol/L
K	4.8				3.5-5.1 mmol/L
BUN	4				6-23 mg/dl
Creatinine	0.61				0.5-1.2 mg/dl
Ph	7.54				7.32-7.43
PCO2	32				38-41 mmHg
HCO3	27				22-29 mmol/L
CK	78				5 to 25 IU/L.
Lipase	23				10-140 U/L
Lactic acid	1.8				0.6- 1.4 mmol/L

**Figure 1:**
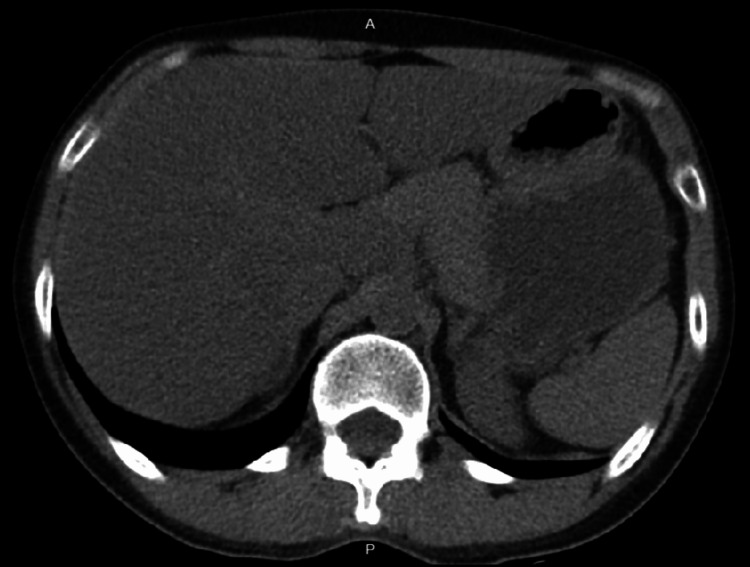
CT abdomen shows fatty infiltration of the liver without CBD dilatation CT: computed tomography; CBD: common bile duct.

CT abdomen and pelvis without contrast revealed fatty infiltration of the liver without common bile duct (CBD) dilatation or other intra-abdominal pathology (Figure 1). A US abdomen revealed a limited right upper quadrant study due to pain, but the liver measured 14.6 cm without CBD dilatation. The patient was given IV fluids with Zofran 4 mg IV and Zosyn 4.5 g IV in the emergency department.

The patient was admitted to the medical floor for close monitoring and further management. IV fluids, Zofran 4mg iv twice daily, proton pump inhibitor 40 mg IV twice daily, thiamine iv once daily, and vitamin B 6 iv once daily, were administered. An acute hepatitis work-up revealed a methyl alcohol level of <0.010. A hepatitis panel, including hepatitis B virus (HBV) surface antigen (HBsAg), antibody to hepatitis A virus, IgM (HAV Ab IgM), and hepatitis C antibody (HCV Ab), were negative, while cytomegalovirus (CMV), Epstein-Barr virus (EBV), and HIV were negative. Salicylate levels were <0.3, acetaminophen levels were <5.0, and anti-smooth muscle antibody (ASMA) and liver-kidney microsomal (LKM) antibodies were negative. GI service was consulted and recommended monitoring of LFTs and supportive treatment. The following day liver enzymes dropped to alkaline phosphatase (ALP): 363 U/L, alanine transaminase (ALT): 319 U/L, aspartate aminotransferase (AST): 1787 U/L, then over the next two days, enzymes dropped to ALP: 209 U/L, ALT: 101 U/L, AST: 168 U/L (Table 1).

Based on the exclusion of other possible causes of acute liver injury, as the patient's blood pressure was maintained throughout their hospitalization and in the emergency department, ischemic (shock) hepatitis was ruled out. Viral acute hepatitis was excluded with a negative virology result. The presence of a normal creatine kinase (CK) level excluded rhabdomyolysis. The non-detected level of ethyl alcohol (EtOH) and the presentation after three days after the last drink, which was not a binge drinker, excluded alcohol-induced hepatitis. The negative specific antibodies have also ruled out female autoimmune hepatitis. With normal imaging, obstructive jaundice with cholestatic hepatitis was ruled out, and no other drug history was reported. As she presented suddenly after consuming a large quantity of marijuana, our diagnosis is likely marijuana-induced acute hepatitis.

## Discussion

Hepatitis is characterized by liver inflammation and is caused by infectious pathogens, medications, autoimmune diseases, environmental toxins, alcohol, or illicit drugs [3]. Hepatic injuries caused by drugs account for 20-40% of fulminant hepatic failures [4]. Recreational marijuana use is one of the most prevalent drug-use behaviors. According to National Surveys on Drug Use and Health, 48.2 million Americans, or approximately 18% of Americans, have used marijuana at least once in 2019 [5]. 

Marijuana is not a single-agent compound but a complex mixture of compounds in variable proportions, including terpenoids, cannabinoids, and flavonoids [6,7]. According to a study conducted on chronic marijuana users to determine chemical changes, 76.9% had transaminitis, 57.7% had hepatomegaly, 73.1% had splenomegaly, and 46.2% had hepatosplenomegaly [1]. A randomized controlled trial conducted by Watkins et al. revealed that healthy individuals who received therapeutic doses of cannabidiol had elevated ALT after 3.5 weeks, consistent with hepatic injury. The effect was consistent regardless of the participant's baseline characteristics [2].

Tetrahydrocannabinol (THC) present in marijuana is associated with the upregulation of fatty acid synthase and acetyl co-enzyme A carboxylase while reducing carnitine palmitoyl transferase, causing lipogenesis in the liver, depleting the cellular adenosine triphosphate (ATP) leading to hepatocyte injury [8]. In up to 25% of patients presenting with marijuana use, liver biopsy results revealed evidence of subclinical injury to hepatocytes [9]. Daily use of cannabis has also been linked to a higher risk of HIV co-infection than cannabis use on an occasional basis [10,11]

Animal studies suggest that cannabinol can have different effects based on the particular receptor activated. Activation of cannabinoid receptor (CB) 2 is found to be hepato-protective, while stimulation of CB 1 is associated with steatogenic and fibrogenic effects. This effect is of much clinical research interest these days for being a possible treatment for hepatic fibrosis [10]. This hepato-protective effect was also found in a population-based study performed by ElTelbany et al., where it was shown that people who use cannabis were 55% less likely to have hepatocellular carcinoma than those who use marijuana [12].

Marijuana-related hepatic injury remains a diagnosis of exclusion after excluding other more prevalent causes, such as acetaminophen toxicity, alcohol-related injury, and viral infections [13]. A marijuana-induced acute hepatic injury diagnosis was made in the presented patient since all pertinent blood tests for alternative causes of hepatic injury were negative. In light of considerable marijuana use, a diagnosis of marijuana-induced acute hepatic injury was made. Based on the severity of the hepatic injury in our patient, only conservative treatment was necessary. Abstinence from marijuana use is generally recommended as a supportive treatment for marijuana-related hepatotoxicity. During the acute phase, N-acetylcysteine (NAC) may be used for its hepatoprotective properties to slow the progression of liver damage [14].

## Conclusions

The use of synthetic marijuana has been linked to hepatic failure in multiple case reports, but natural marijuana has not yet been proven to cause fulminant liver toxicity. Therefore, the differential diagnosis of hepatic injury in drug abusers must include marijuana as well as classical viral and alcoholic hepatitis. A mechanism of action must be identified. However, definitive determination of the mechanism lags behind that of cannabimimetic agents that act on liver CB receptors.
